# An in vitro method to keep human aortic tissue sections functionally and structurally intact

**DOI:** 10.1038/s41598-018-26549-4

**Published:** 2018-05-25

**Authors:** Jorn P. Meekel, Menno E. Groeneveld, Natalija Bogunovic, Niels Keekstra, René J. P. Musters, Behrouz Zandieh-Doulabi, Gerard Pals, Dimitra Micha, Hans W. M. Niessen, Arno M. Wiersema, Jur K. Kievit, Arjan W. J. Hoksbergen, Willem Wisselink, Jan D. Blankensteijn, Kak K. Yeung

**Affiliations:** 1 VU University Medical Center, Department of Vascular Surgery, Amsterdam, The Netherlands; 2 VU University Medical Center, Amsterdam Cardiovascular Sciences, Department of Physiology, Amsterdam, The Netherlands; 3 VU University Medical Center, Department of Clinical Genetics, Amsterdam, The Netherlands; 4 VU University Medical Center, Department of Pathology, Amsterdam, The Netherlands; 5 Westfriesgasthuis, Department of Vascular Surgery, Hoorn, The Netherlands

## Abstract

The pathophysiology of aortic aneurysms (AA) is far from being understood. One reason for this lack of understanding is basic research being constrained to fixated cells or isolated cell cultures, by which cell-to-cell and cell-to-matrix communications are missed. We present a new, *in vitro* method for extended preservation of aortic wall sections to study pathophysiological processes. Intraoperatively harvested, live aortic specimens were cut into 150 μm sections and cultured. Viability was quantified up to 92 days using immunofluorescence. Cell types were characterized using immunostaining. After 14 days, individual cells of enzymatically digested tissues were examined for cell type and viability. Analysis of AA sections (N = 8) showed a viability of 40% at 7 days and smooth muscle cells, leukocytes, and macrophages were observed. Protocol optimization (N = 4) showed higher stable viability at day 62 and proliferation of new cells at day 92. Digested tissues showed different cell types and a viability up to 75% at day 14. Aortic tissue viability can be preserved until at least 62 days after harvesting. Cultured tissues can be digested into viable single cells for additional techniques. Present protocol provides an appropriate *ex vivo* setting to discover and study pathways and mechanisms in cultured human aneurysmal aortic tissue.

## Introduction

Aortic aneurysm (AA) is a common health problem, which is associated with high mortality rates in the event of a rupture. This unpredictable life-threatening rupture leads to rapid aortic exsanguination into thorax, abdominal cavity or retroperitoneum^1–3^. To date, studies investigating the pathophysiology of AA have produced inconclusive results concerning the underlying mechanisms. Although different meaningful disease models exist, the lack of a functionally and structurally intact live aortic human tissue model which can bioactively be stimulated *ex vivo*, has posed severe limitations to the study of AA in understanding the pathophysiological mechanism which has restricted the identification of therapeutic targets and the development of efficient therapy^4^.

It has been observed previously that an increase of AA diameter occurs in relation to the transformation of the aortic vessel wall composition. Part of this transformation is a combination of vascular smooth muscle cell (SMC) loss and inflammation and degeneration of extracellular matrix (ECM). These changes are suggested to play a role in the pathogenesis of AA, but mechanisms contributing to these processes, are still poorly understood^5,6^.

Although numerous *in vivo* animal models to study AA exist, investigation of human material is limited to the use of fixated tissue or isolated cell cultures^7–12^. Such approaches, however, have failed to retain the complex organ function of the aorta, which is based on cell-cell interactions, communication with ECM, immune cell infiltration and constant tissue remodelling^13–16^.

Unfortunately, fixated aortic tissue only provides a snapshot into the microstructural characteristics of the diseased tissue, and isolated cultured aortic cells cannot reproduce the complexity of cell-ECM interactions and live pathways. Additionally, usage of animal models is costly, ethically and technically challenging and animal models do not completely resemble the human conditions, as the aortic aneurysms need to be created in the animal model.

Hence, there is a need to provide a new *in vitro* model to study the pathophysiological processes, which are involved in human AA. Live sectioning and preservation of human (aneurysmal) aortic tissue can lead to improvement of the understanding of AA pathogenesis. In this study, we present an innovative method for the investigation of human AA pathophysiology by preserving tissue viability and cellular organization of human aneurysmal tissue *ex vivo* for several weeks.

## Methods

### Human tissues

The present study was approved by the Medical Ethical Committee of the VU University Medical Center (VUmc) Amsterdam. Informed consent files were signed prior to surgery. In case of acute surgery for ruptured aneurysm repair, delayed informed consent was received in the postoperative period as soon as permitted by the health situation of the patients. All experiments were performed in accordance with relevant guidelines and regulations.

**Figure 1:**
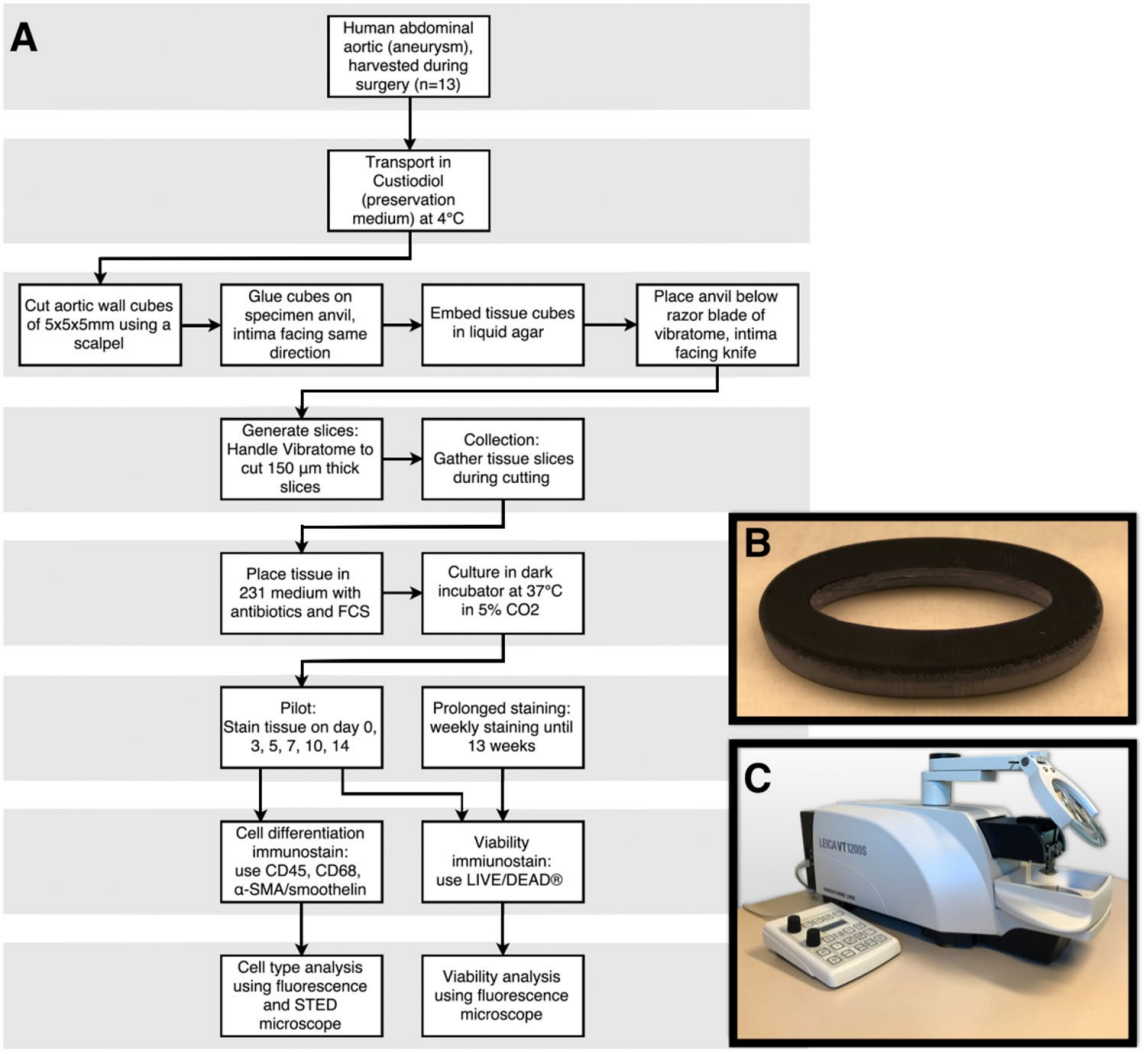
Overview of experimental setup. (**A)** Flowchart of protocol: vascular tissue cubes were cut into sections using a vibrating blade microtome (Leica VT1200S). Tissue sections were cultured in supplemented culture medium in 24-well plates in a dark humidified atmosphere at 37 °C in 5% CO_2_. (**B)** Photograph of self-designed 3D-printed mould in which tissue cubes are fixed in agarose. (**C**) Photograph of vibratome Leica VT1200S, used for tissue sectioning. α-SMA indicates alpha smooth muscle actin; FCS, fetal calf serum and STED, stimulated emission depletion.

Human aortic tissue was collected from the operating theatre in two hospitals (VUmc, Amsterdam, the Netherlands and Westfriesgasthuis, Hoorn, the Netherlands) during open abdominal AA (AAA) surgery (N = 9). Additional vascular tissues were harvested from human non-aortic anatomical sites (N = 4): external iliac artery punch and renal artery. Using nine consecutively harvested human vascular tissues (N[AAA] = 5 and N[additional vascular tissues] = 4), a pilot study with primary viability analysis until 14 days was performed. These results were evaluated to further develop the protocol with minor improvements. Subsequently, new AAA tissue (N = 4) was harvested and subjected to the new protocol to evaluate the maximum life span of human AAA tissue sections *ex vivo* (Fig. 1A).

### Transport and vascular section preparation

Directly after harvesting tissue at the operating theatre, the vascular tissue was submerged in Custodiol (Dr. Franz Köhler Chemie GmbH, Bensheim, Germany) at 4 °Celsius. The tubes containing tissue were transported on ice to the vibratome (Leica VT1200 S, Leica Biosystems, Nussloch, Germany). Vascular cubes of 5 × 5 × 5 mm were cut using a scalpel. These cubes contained intima, media and adventitia. Cubes were then glued on a specimen anvil using Roti Coll superglue (Carl Roth GmbH + Co. KG, Karlsruhe, Germany). The intimal layer of the cubes was facing the knife of the vibratome, to ensure that after cutting, aortic sections were containing all layers. Fixed cubes were embedded in agarose gel solution (1 g agarose in 50 mL 0.5× TAE buffer, UltraPure™ Agarose, Thermo Fisher Scientific Inc., Waltham, MA, USA) using a self-designed 3D-printed mould (Fig. 1B).

After cooling down and thereby stiffening of agarose gel, the mould was removed. The entirety of anvil, tissue and agarose was immersed in a small reservoir filled with Custodiol at 4 °Celsius. The reservoir was placed under the razor blade (Croma Stabil, Feintechnik GmbH, Eisfeld, Germany) of the vibratome (Fig. 1C). Cutting of sections containing intima, media and adventitia was performed. The first three consecutive sliced sections were discarded, since these often macroscopically did not contain all layers of the vascular wall and lower viability in these sections was to be expected. From earlier (unpublished) pilot experiments, we determined that the best viability results were acquired by cutting 150-μm-thick sections with the vibratome at a speed of 0.08 mm/s with a horizontal amplitude of the razor blade fixed at 2.65 mm. For the development of the present protocol, live bovine tissues were firstly used to set up the optimum settings for the vibratome. Abdominal bovine aorta was obtained from an abattoir (Abattoir Amsterdam B.V, Amsterdam, the Netherlands) directly after euthanization.

### Tissue preservation

Immediately after sectioning with the vibratome, tissue sections were transferred in culture medium. Tissue sections were conserved in 24 wells plates, each well filled with 500 μl of one of two different media; either Ham’s F10 (Ham’s F10 Nutrient Mix, Gibco, Life Technologies, Carlsbad, CA, USA) supplemented with Penicillin-Streptomycin (PS, 25,000 U, Gibco, Life Technologies, Carlsbad, CA, USA) and 10% Fetal Bovine Serum (FBS, Gibco, Life Technologies, Carlsbad, CA, USA) or M231 (Medium 231, Smooth Muscle Cell medium, Gibco, Life Technologies, Carlsbad, CA, USA) supplemented with PS (25,000 U) and 5% Smooth Muscle Growth Supplement (SMGS, Gibco, Life Technologies, Carlsbad, CA, USA). Both supplemented culture media (Ham’s F10 and M231) of the first 4 consecutively harvested tissues were compared to determine best outcome on tissue viability. Sections were cultured in wells plates in a dark humidified atmosphere at 37 °Celsius in 5% CO_2_ and culture media were replaced daily.

### Immunofluorescence staining: viability and characterization of cell types

*Viability* was examined by immunofluorescence staining at day 0, 3, 5, 7, 10 and 14 for primary analysis (N = 9). After protocol optimization, the newly harvested tissues (N = 4) were kept viable as long as possible and were examined monthly by the immunostaining for viability. For analysis, tissues were removed from the culture medium and washed in PBS solution for two minutes. Subsequently, the tissue was incubated in chamber slides for 30 minutes in 300 µl of 0.04% calcein acetoxymethyl (calcein AM) and 0.16% ethidium homodimer-1 (EthD-1) solution (LIVE/DEAD® Viability/Cytotoxicity Kit and PBS solution, Life Technologies, Carlsbad, CA, USA). LIVE/DEAD® Kit stains live and dead cells by accordingly indicating intracellular esterase activity or loss of plasma membrane integrity. Cell-permeant nonfluorescent calcein AM is enzymatically converted to intensely fluorescent calcein (green) by live cell intracellular esterases, which hydrolyse acetoxymethyl ester. EthD-1 becomes fluorescent (red) in cell nuclei upon binding to nucleic acids after entering through the damaged cell membrane of dead cells. Given that live cells have intact cell membranes, transmembrane passage of EthD-1 in live cells does not take place.

Earlier, in feasibility tissues, staining with anthracycline derivative 1% DRAQ7 dye (DRAQ7™, Cell Signaling Technology, Inc., Danvers, MA, USA) in combination with 1% Hoechst (Hoechst 33342™, Cell Signaling Technology, Inc., Danvers, MA, USA) and DNA dye 1% DRAQ5™ (DRAQ5™, Cell Signaling Technology, Inc., Danvers, MA, USA) were used to monitor cell death. However, the latter stained only the upper layer of the tissue, while LIVE/DEAD® Kit stained through the whole thickness of the aortic tissue; making quantification of the live and dead cells possible. In order to characterise cell type composition of the tissue, SMC, leukocytes and macrophages were subjected to immunofluorescence staining against α-SMA/smoothelin, CD45, CD68 respectively. First, tissue was washed twice with 300 µl of PBS and fixated in 300 µl of 4% formaldehyde solution (Sigma-Aldrich, St. Louis, MO, USA) for 20 minutes. Fixated tissue was subsequently washed three times for five minutes in 300 µl of 0.05% PBST (Tween and PBS solution, Sigma-Aldrich, St. Louis, MO, USA) and submerged in a 0.2% solution of Triton (Triton x-100 and PBS solution, Sigma-Aldrich, St. Louis, MO, USA) for twenty minutes and rinsed with a 1.0% BSA solution (Bovine Serum Albumine and PBS solution, Vector Laboratories, Inc, Burlingame, CA, USA). After three PBST washing steps of five minutes, the tissue was incubated overnight in 2% Monoclonal Mouse Anti-Human Smooth Muscle Actin solution at dilution; (DAKO, Glostrup, Denmark). Hereafter, five washing steps of five minutes using PBST, were followed by one hour incubation of 1% Anti-Mouse Alexa Fluor 647 secondary antibody (Thermo Fisher Scientific Inc.). After five PBST washing steps, overnight incubation using 2% Polyclonal Rabbit Anti-Human Primary Smoothelin Antibody (H-300, Santa Cruz Biotechnology, Dallas, TX, USA) was performed. Five PBST washing steps were then followed by incubation of one hour using 1% Anti-Rabbit Alexa Fluor 488 secondary antibody (Thermo Fisher Scientific Inc.).

In additional slides, leukocytes were stained with 2% Monoclonal Mouse Anti-human CD45 (N = 1, DAKO, Glostrup, Denmark) and macrophages were stained with 2% Monoclonal Mouse Anti-human CD68 (N = 1, DAKO, Glostrup, Denmark) in fixated coupes and after washing with PBST (five steps) followed by incubation of one hour using 1% Anti-Mouse Alexa Fluor 647 secondary antibody (Thermo Fisher Scientific Inc.). Where overnight staining was done at 4 °Celsius, the remainder steps were performed at room temperature. Aforementioned α-SMA/smoothelin, CD45 and CD68 stainings were followed by 15 minutes staining for 2% DAPI (4′,6-Diamidino-2-Phenylindole, Dihydrochloride, Thermo Fisher Scientific Inc.) to show cell nuclei and tissue was washed with PBST twice and once with PBS solution at room temperature. Slides with tissue were mounted with VECTASHIELD® Antifade Mounting Medium (Vector Laboratories, Inc, Burlingame, CA, USA) and closed using coverslips. As negative controls for α-SMA/Smoothelin, CD45 and CD68, tissue sections were used in which the primary antibody was omitted from the staining procedure.

In order to assess wall composition over time, aortic tissue sections of one AAA patient were cultured until 14 days in daily replaced supplemented M231 culture medium. After 0, 7 and 14 days, different tissue sections were removed from the culture medium and snap-frozen for analysis. Therefore, the sections were immunostained for smoothelin, as a marker of mature SMC, and DAPI according to above described protocol. Additionally, phalloidin (Rhodamine-phalloidin, Thermo Fisher Scientific Inc.) immunostaining was performed simultaneously with DAPI immunostaining, to present all aortic wall cells and their relation within the tissue sections. Again, after immunostaining, tissues were washed, mounted with VECTASHIELD® and slides were closed using coverslips.

### Vascular enzymatic tissue digestion for analysis with individual cells

Multiple tissue sections of two AAA patients were separately submerged in 1 mg/ml collagenase type II (Worthington, Lakewood, NJ, USA) and M231 in tubes. These were placed in a rotator in a dark atmosphere at 37 °Celsius for 3 hours. The mixture was then filtrated through a 100 μM gauze and centrifuged at 300 g for 10 minutes. Supernatant was removed and cell pallet was resuspended in 500 μL of M231. These cells were used for further qualitative immunofluorescence analysis and count and viability assay.

### Immunofluorescence staining in digested cells

After primary analysis at day 0, tissues were digested at day 7 and 14 as described above, to quantitatively analyze cell viability and qualitatively analyze characteristics in individual cells. Cells were stained with LIVE/DEAD® Kit, using the same staining protocol as for tissues.

### Analysis of immunofluorescence staining

Viability examination of sections was performed using Zeiss Axiovert 200 M Marianas™ digital imaging inverted microscope system. The set-up was provided with a non-stepper-motor (z-axis increments: 0.1 µm) and a filter turret with individual filter blocks for fluorescein isothiocyanate (FITC), a pair of cyanine dyes (CY5 and CY3), aminomethylcoumarin acetate (AMCA) and a differential interference contrast (DIC) brightfield cube. FITC and CY3 were handled to respectively demonstrate live (calcein) and dead (EthD-1) cells. Imaging was performed using a 16-bit, cooled charge-coupled device camera (Cooke Sensicam SVGA, Cooke Co., Tonawanda, NY, USA). Aforementioned set-up was connected to Slidebook™ (Slidebook v. 5.5 software, Intelligent Imaging Innovations, Inc., Denver, CO, USA) to control hardware and to view and process images. Zeiss air objective lenses at magnifications of 2.5× (for quantitative overview images), 10× and 40× (for detailed higher resolution images and 3D stacks) and 63× (oil objective lens to study individual cells) were used to obtain images. Unspecific background and disproportional intensity staining was corrected for by Slidebook software. The viability proportion of tissue sections was calculated by dividing calcein intensity in square micron by the sum of calcein and EthD-1 in square micron.

Analysis of different cell types was again performed using Zeiss Axiovert 200 M Marianas™. Depending on choice of different secondary antibodies CY5, CY3, FITC and DAPI were utilized. Magnifications of 2.5×, 10× and 40× were used for analysis. For characterization of cell type, shutter speeds were based on primary antibody positive stained tissues, while use of Renormalize Button in Slidebook™ was based on negative controls (without primary antibody). To obtain super resolution images a Confocal Laser Scanning Microscope Leica TCS SP8 (Leica Microsystems, Mannheim, Germany) was used. Regions of interest in images previously captured with fluorescence microscope were reviewed using 40× and 63× Leica oil-immersion lenses. Unfortunately, photobleaching of calcein interfered with visualization of live cells. Therefore, for live and dead quantification solely fluorescence microscopy was used. LAS-X software was used to analyse images of cell type differentiation. Excitation/emission spectra used for secondary antibodies (Alexa Fluor 488, Alexa Fluor 555, Alexa Fluor 647) were chosen out of identically named pre-set excitation/emission spectra in Leica LAS-X.

Aortic wall composition analysis was performed using Nikon A1R (Nikon Instruments, Tokyo, Japan) confocal microscope with 40× oil objective. Using NIS-Elements C Software (Nikon Instruments), super high-resolution (5120 × 5120 pixels) intima-to-adventitia stitched images of 5 × 5 fields of view. Excitation/emission spectra used to capture fluorescent signal of phalloidin (Alexa Fluor 546), and secondary antibody of smoothelin (Alexa Fluor 647) were chosen out of pre-set excitation/emission spectra in NIS-Elements C. Fluorescence levels of smoothelin and phalloidin were quantified using FIJI/ImageJ (v1.0. National Institutes of Health, Bethesda, MD, USA). Following background subtraction, masks of selected tissue areas were created and quantified. Outcomes were corrected for number of cells using DAPI count.

### Count and viability assay following tissue digestion

On day 14, individual cell viability assay was performed by the Muse Count & Viability reagent (Millipore, Billerica, MA, USA) following the manufacturer’s protocols. Harvested cells in M231 (50 μL) were added to 450 μL Count & Viability reagent (Millipore). The system was gated for viability and exclusion of both cellular debris and cell clusters, which was based on cultured commercially available smooth muscle cells of a 31-year old healthy male (Thermo Fisher Scientific Inc., Waltham, MA, USA). The results were analyzed with Muse Count & Viability software module.

### Proof of concept: *ex vivo tissue* stimulation and quantification of gene expression by qPCR

Live aortic tissues of AAA patients (N = 8) were sectioned according to above described protocol. Complete M231 culture medium (including PS and SMGS) was supplemented with TGF-β (5 ng/ml, BioVision, Milpitas, CA) to stimulate the tissue sections, since dysregulation of TGF-β is often described in literature, as a key player an in the development and progression of AAA. Culturing of sections was performed in complete M231 medium with (stimulated) and without TGF-β (non-stimulated) supplement in a dark humidified atmosphere at 37 °Celsius in 5% CO_2_. Culture medium was replaced daily to ensure constant TGF-β stimulation. After 7 days, both stimulated and non-stimulated tissues were rinsed in PBS and directly snap-frozen. Tissue pieces were stored at −80 °Celsius until analysis.

AAA patient pooled (N = 8) stimulated and non-stimulated, tissue sections were homogenized apart from each other in two 2.0 mL eppendorfs in 300 µL lysis buffer (Zymo Research, Irvine, CA, U.S.A.). Total RNA of both eppendorfs was isolated using Quick-RNA™ MiniPrep kit (Zymo Research). Synthesis of complementary DNA was performed in a reverse transcription reaction using VILO kit (Thermo Fisher Scientific Inc.). Quantitative PCR (qPCR) was performed to analyze gene expression. *YWHAZ* was used as housekeeping gene. By use of LightCycler® SYBR Green I Master (Roche Applied Science, Penzberg, Germany) gene expression was analyzed by the LightCycler® 480 Instrument II (Roche Applied Science). Potential differences in expression of interleukin 6 *(IL6)*, calponin 1 *(CNN1)*, transforming growth factor beta 1 *(TGFB1)*, monocyte chemotactic protein 1 *(MCP1)*, transforming growth factor beta receptor 1 *(TGFBR1)*, smoothelin *(SMTN)*, intercellular adhesion molecule 3 *(ICAM3)*, matrix metalloproteinase-2 *(MMP2)*, alpha smooth muscle actin 2 *(ACTA2)*, tumor necrosis factor *(TNF)*, protein tyrosine phosphatase, receptor type, C *(PTPRC)*, antigen KI-67 *(Ki67)*, intercellular adhesion molecule 1 *(ICAM1)*, interleukin 8 *(IL8 or CXCL8)* and matrix metalloproteinase-9 *(MMP9)* in stimulated and non-stimulated AAA tissue sections were evaluated (the entire list of corresponding NCBI Reference Sequence Database codes and forward and reverse primer sequences can be found as Supplementary Table S1). RNA expression was shown as a ratio; relative expressions based on non-stimulated tissue sections was calculated after seven days culturing *ex vivo*.

### Statistical analysis

The data was analyzed with SPSS Statistics 22.0 (IBM Corporation, Armonk, NY, USA). Measurements of viability are shown in box-plots. Wilcoxon signed-rank test was performed to compare viability of corresponding tissue cultured in different media (Ham’s F10 and M231). Comparisons of viability in the first nine consecutive patient sample on multiple time points were made by repeated measures of analysis of variance (one-way Anova) using the Bonferroni correction for multiple-comparison testing. P-value of < 0.05 is considered statistically significant. Muse Count & Viability software module showed percentage of viable single cells of enzymatically digested tissues.

### Data availability

The datasets generated during and analyzed during the current study are available from the corresponding author on reasonable request.

## Results

Primary viability analysis was performed in 9 vascular specimens. After a few adjustments of the protocol (i.e. life-span optimization during sectioning and culturing of the tissues), maximum life endurance of live aortic *ex vivo* tissues was examined in 4 additional aortic tissues. Viability staining showed no difference in viability between cultured tissues (N = 4) in Ham’s F10 or M231 culture media (α = 0.465). Since no significant difference was observed between tissue viability cultured in different media, the mean outcomes of tissues in either Ham’s F10 or M231 per day per corresponding donor were used for further analysis.

**Figure 2:**
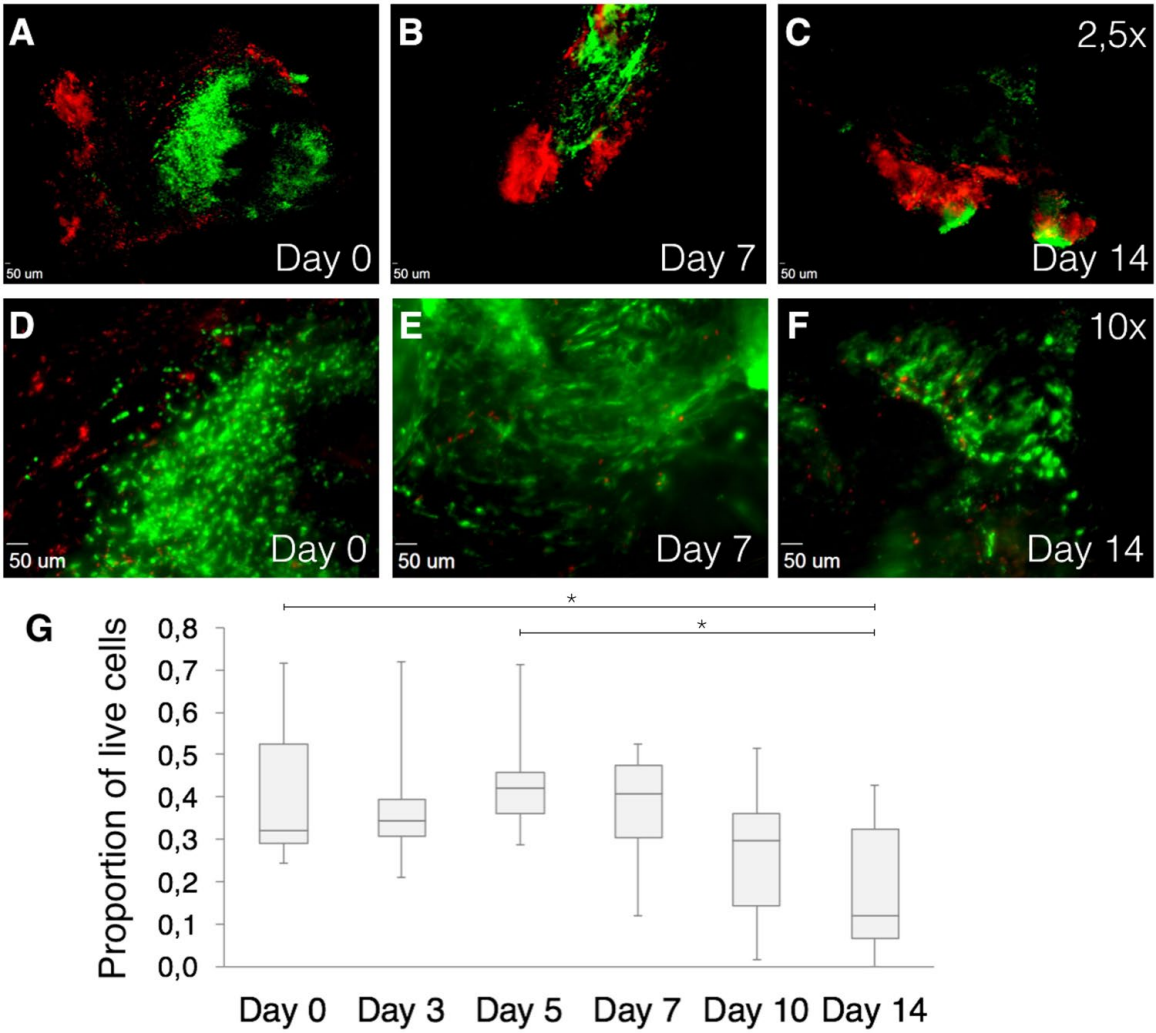
Immunofluorescence images using Zeiss Axiovert 200 M Marianas™ Microscope. Cells stained with LIVE/DEAD® Viability/Cytotoxicity Kit. (**A**–**B**) 2.5× magnification of cultured tissues at day 0, 7 and 14, respectively. (**D**–**F**) 10× magnification of cultured tissues at day 0, 7 and 14, respectively. Green fluorescence shows live cells, while red fluorescence indicates dead nuclei. Live cells are mainly located central to tissue. (**G)** Quantification of live human cells in harvested vascular tissues of distinct patients (N = 9). Box plots show proportions of square micron of green fluorescence divided by the sum of green and red fluorescence. *P < 0.05 compared with other time points using ANOVA with Bonferroni test.

**Figure 3:**
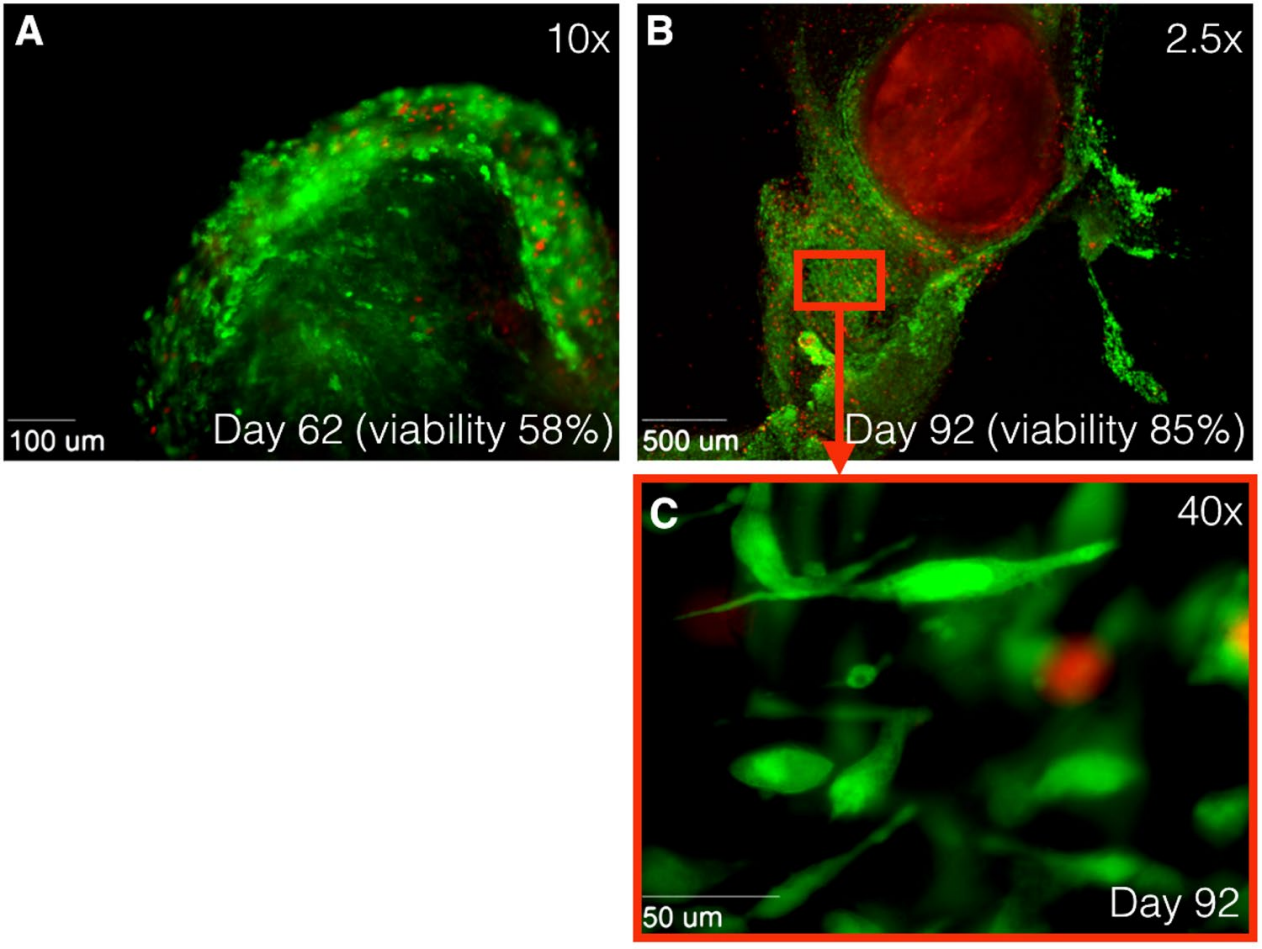
Immunofluorescence images at 10× magnification using Zeiss Axiovert 200 M Marianas™ Microscope. Human tissue stained with LIVE/DEAD® Viability/Cytotoxicity Kit. Green fluorescence shows live cells, while red fluorescence indicates dead nuclei. (**A)** Alive tissue at day 62 after harvesting. Tissue viability of 58%. (**B)** Alive tissue at day 62 after harvesting at 2.5× magnification and (**C**) at 40× magnification. Outgrowth of new cells is observed after 92 days, while original tissue shows only staining of EthD-1 (dead cells). Tissue viability of 85%. EthD-1 indicates ethdium homodimer-1.

Qualitative evaluation of all images of the first 9 vascular tissues showed that dead cells were located mostly on the surface of the tissue, while live cells were mainly localized in the center of vascular tissue sections (Fig. 2A–F). Figure 2G shows tissue viability during the experiments; human tissues at day 0 showed a mean viability proportion of 0.42 (N = 9, SD: 0.16). Consecutively, mean viability proportion was 0, 38 (SD: 0.15) for day 3; 0, 43 (SD: 0.12) for day 5; 0, 38 (SD: 0.13) for day 7; 0, 27 (SD: 0.17) for day 10; and 0, 18 (SD: 0.15) for day 14. There was a significant difference in viability proportion between day 0 and day 14 (P = 0.026) and between day 5 and day 14 (P = 0.015). AAA tissue viability at day 0 was 0.42 (N = 5, SD: 0.2), which was almost similar to the non-aortic-tissue viability at day 0, being 0.41 (N = 4, SD: 0.14). Small protocol adjustments were implemented, including pre-sectioning removal of calcium depositions, warmed (37 °Celsius) tissue section transport, immunostaining within culture medium and shorter tissue-out-of-incubator times. After implementing the new protocol, an improved tissue viability proportion of 0.58 after 62 days was observed (Fig. 3A) in AAA tissue. After 92 culturing days (viability proportion: 0.85), outgrowth of many new cells around the initial tissue was observed, but no viability in the original tissue was seen (Fig. 3B,C).

### Immunofluorescence: smooth muscle cells, leucocytes and macrophages, and aortic wall composition

**Figure 4:**
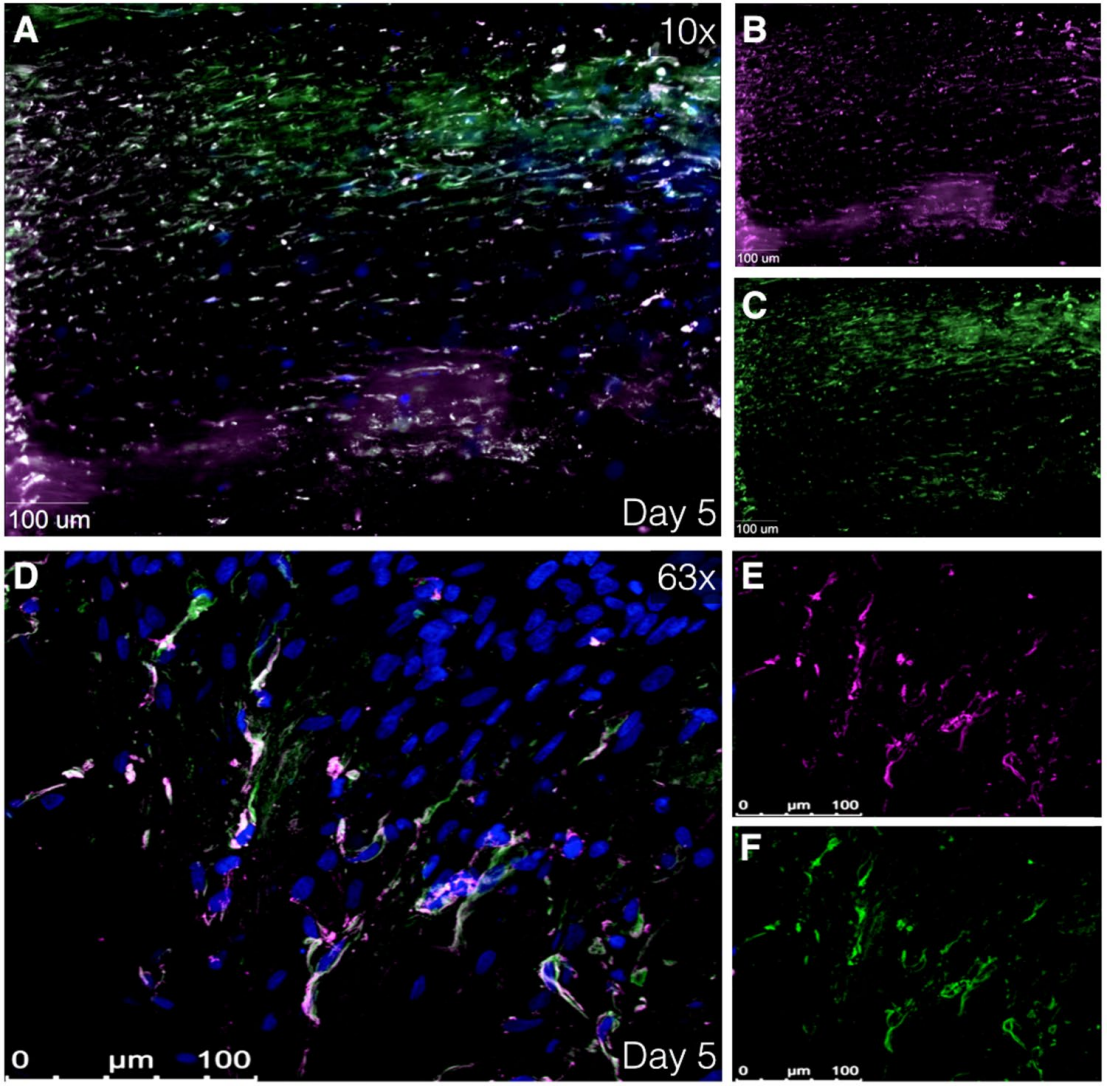
Immunofluorescence images showing smooth muscle cells. (**A**–**C**) Immunofluorescence images at 40× magnification using using Zeiss Axiovert 200 M Marianas™ Microscope at day 5 after harvesting. (**D**–**F)** Immunofluorescence image at 63× magnification using super resolution Confocal Laser Scanning Microscope Leica TCS SP8. Human tissue stained with α-SMA and smoothelin (smooth muscle cell markers) at day 5 after harvesting. (**A**,**D)** Merged image of α-SMA (purple), smoothelin (green) and DAPI (blue). (**B**,**E)** Isolated α-SMA staining. (**C**,**F)** Isolated smoothelin staining. α-SMA indicates alpha smooth muscle actin and DAPI, 4′,6-diamidino-2-phenylindole.

**Figure 5:**
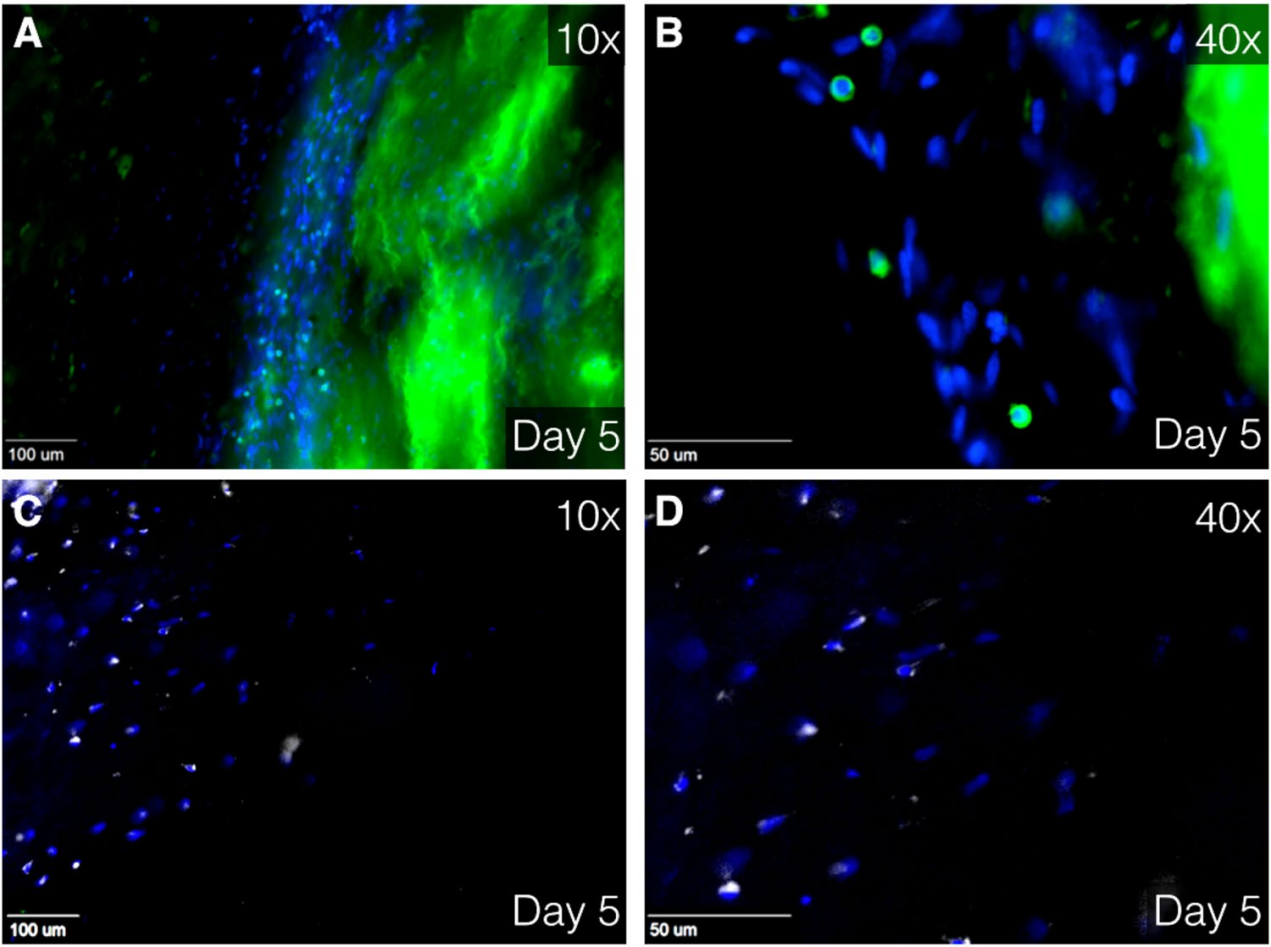
Immunofluorescence image at 10× (**A**,**C**) and 40× (**B**,**D**) magnification using Zeiss Axiovert 200 M Marianas™ Microscope. Human tissue stained with DAPI (blue), CD45 (green, **A**,**B**) and CD68 (white, **C**,**D**) Green staining at the right side (**A**,**B**) is due to unintentional fluorescence of elastin. CD indicates cluster of differentiation and DAPI, 4′,6-diamidino-2-phenylindole.

Given that primary analysis showed best viability outcomes on day 5 and 7, these days were designated to perform further experiments. Cells with a similar shape to SMC were observed during analysis of viability images using LIVE/DEAD® Kit (Fig. 3C). Digital imaging microscope analysis of immunofluorescence staining showed cells with colocalized α-SMA and smoothelin, early and late SMC marker, respectively (Fig. 4A–C), again with aforementioned characteristic SMC shape. Additionally, confocal scanning laser microscopy was utilized to gain high quality images, which confirmed the presence smooth muscle cells (Fig. 4D–F). Moreover, fixated immunostaining for CD45 and CD68 showed leukocytes and macrophages, respectively (Fig. 5A–D). By retrospectively analyzing the images of tissues immunostained with LIVE/DEAD® Kit, the morphological form of live leukocytes could be recognized.

**Figure 6:**
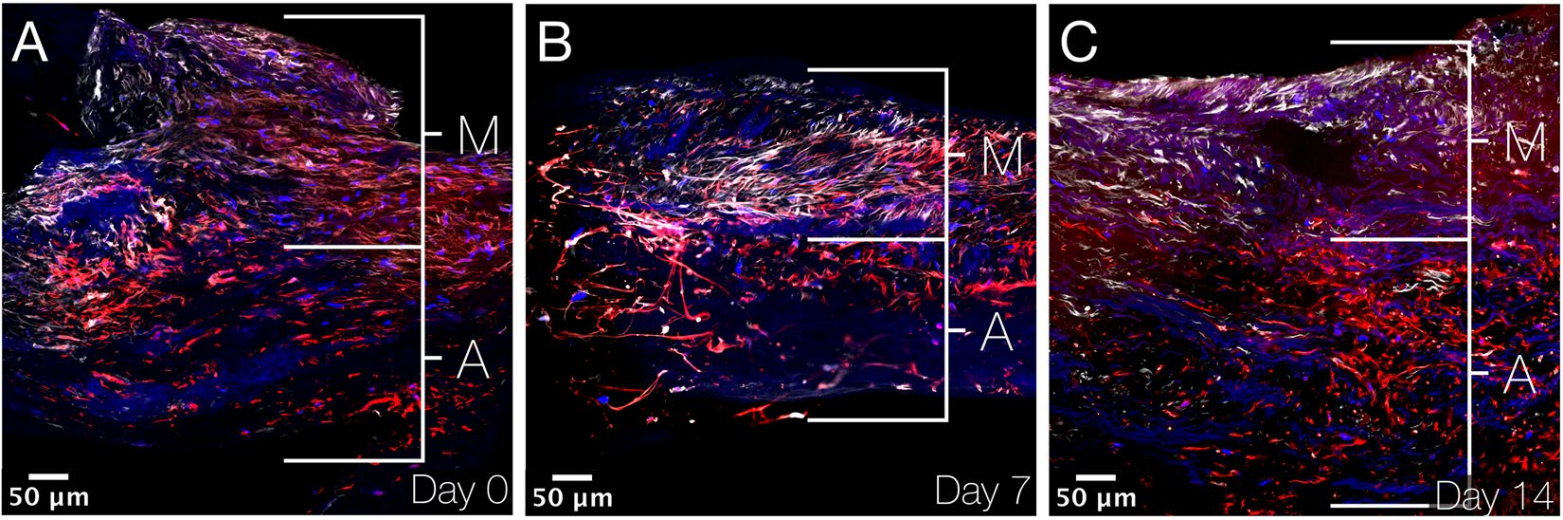
Immunofluorescence images showing aneurysmal aortic wall sections. (**A–C)** XY stitched images at 40× magnification using Nikon A1R confocal microscope on day 0, 7 and 14 after culturing. Smooth muscle cells are immunostained by smoothelin (white), actin (found in practically all eukaryotic cells) by phalloidin (red) and cell nuclei by DAPI (blue). Smooth muscle cells are found in the media and other cells (mainly fibroblasts) in the adventitia. M indicates media; A, adventitia and DAPI, 4′,6-diamidino-2-phenylindole.

Stitched overview images of the aneurysmal aortic wall show smooth muscle cells (immunostained by smoothelin) in the upper part of the tissues at day 0, 7 and 14 after culturing (Fig. 6). Phalloidin immunostaining shows all cells of the aortic wall. Thereby, co-staining of both smoothelin and phalloidin indicates the tunica media (mainly consisting of smooth muscle cells), while in underlying tissue only expression of phalloidin is observed, indicating the tunica adventitia (mainly consisting of fibroblasts). This distinction between tunica media and tunica adventitia is observed at day 0, 7 and 14. Fluorescence quantification of smoothelin per nucleus shows a fold increase of 1.6 and 2.3 for day 7 and day 14, respectively. Fluorescence quantification of phalloidin per nucleus shows a fold increase of 4.8 and 5.5 for day 7 and day 14, respectively.

### Outcome of enzymatic digestion of the aortic tissue: individual cell analysis

**Figure 7:**
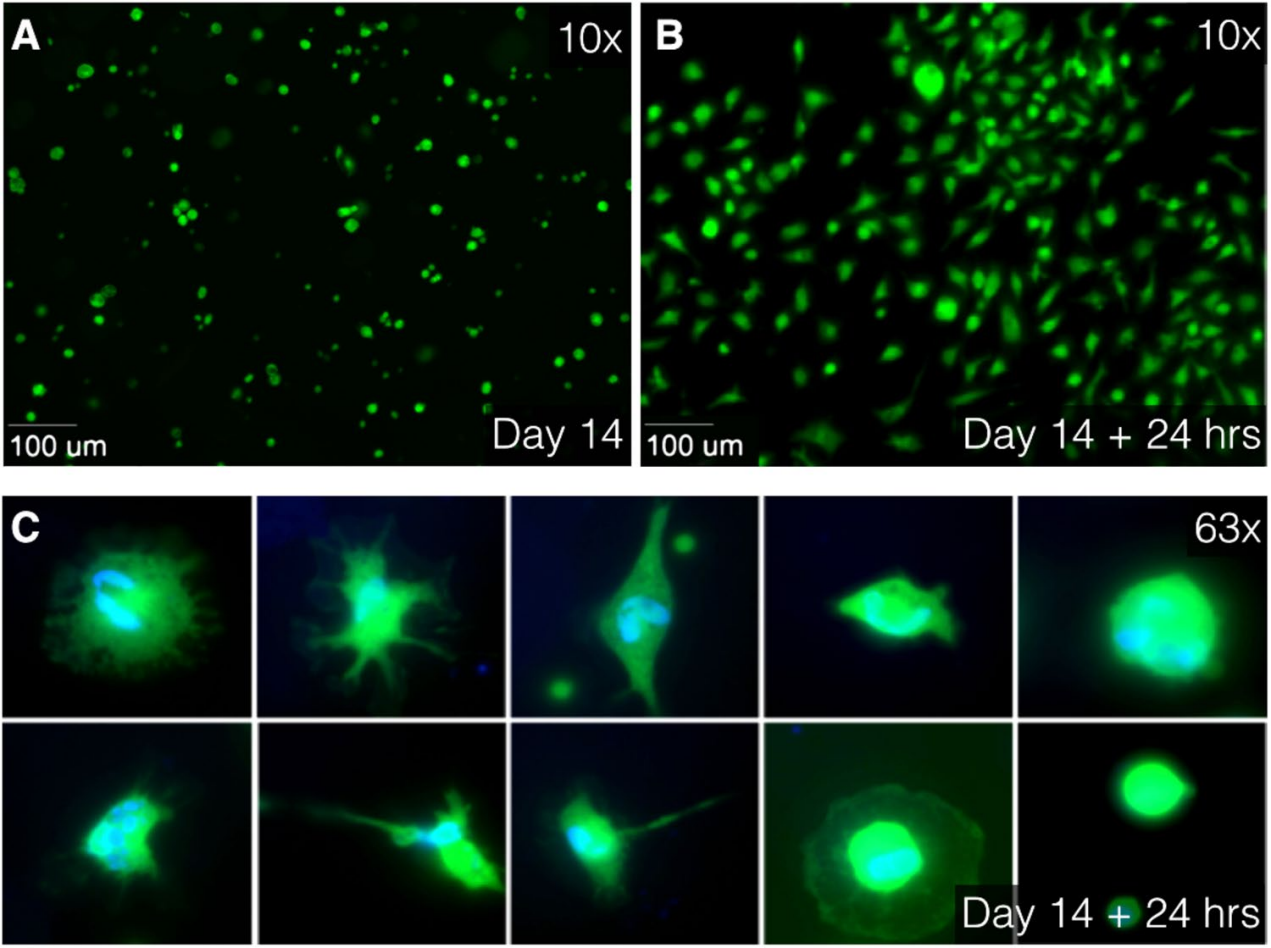
Immunofluorescence images at 10× (**A**,**B**) and 63× oil (**C**) magnification using Zeiss Axiovert 200 M Marianas™ Microscope. (**A**) After 14 days of culturing, tissues were enzymatically digested using collagenase. Live separate cells, stained with Calcein AM (green), float as round cells in culture medium. (**B)** After 24 hours of additional culturing, the cells were attached to the slide. (**C)** Different cell type characteristics and differences in cell nuclei are observed in attached cells. Live cytoplasm is stained with Calcein AM (green) and cell nuclei are stained with DAPI (blue). AM indicates acetoxymethyl and DAPI, 4′,6-diamidino-2-phenylindole.

Tissues of AAA patients (N = 2) at day 14 in culture were enzymatically digested. Fluorescent calcein was observed in cytoplasm of enzymatically-digested cells, surrounding the nucleus. Separate cells attached to the slide after enzymatic digestion with collagenase. Different cell shapes and nuclei were observed in the individual cells (Fig. 7). Digested tissue sections showed viability of 0.75 (in 950.000 cells) in one patient and viability of 0.83 (in 800.000 cells) in the other patient.

### RNA expression results of stimulated and non-stimulated aortic tissue sections

**Figure 8:**
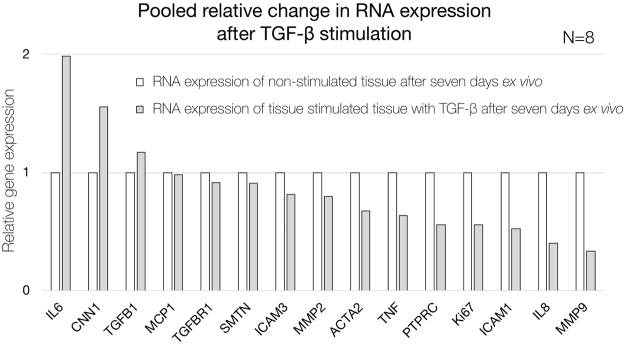
Quantification of gene expression of pooled non-stimulated (white) and TGF-β-stimulated (grey) AAA patient tissue sections after seven days *ex vivo*. Relative expression is shown as ratio based on non-stimulated tissue sections. *IL6* indicates interleukin 6; *CNN1*, calponin 1; *TGFB1*, transforming growth factor beta 1; *MCP1*, monocyte chemotactic protein 1; *TFBR1*, transforming growth factor beta receptor 1; *SMTN*, smoothelin; *ICAM3*, intercellular adhesion molecule 3; *MMP*2, matrix metalloproteinase-2; *ACTA2*, alpha smooth muscle actin 2; *TNF*, tumor necrosis factor; *PTPRC*, protein tyrosine phosphatase, receptor type, C; *Ki67*, antigen KI-67; *ICAM1*, intercellular adhesion molecule 1; *IL8 (or CXCL8)*, interleukin 8 and *MMP9*, matrix metalloproteinase-9.

Tissue sections of eight AAA patients were subjected to stimulation with TGF-β for 7 days. Control tissue sections of the same patients were cultured without TGF-β stimulation. Pooled RNA analysis of stimulated tissue sections was relatively related to non-stimulated tissue sections. In this feasibility analysis, elevated relative expression of interleukin 6 *(IL6)*, calponin 1 *(CNN1)* and transforming growth factor beta 1 *(TGFB1)* was observed in the pool of TGF-β stimulated tissue sections, while the remainder of the studied genes showed a decrease in expression (Fig. 8).

## Discussion

The underlying mechanisms of the development, progression, and rupture of AA are still poorly understood. Prior studies on AA were performed in animal models, fixated human tissue, or isolated human cell cultures. An *in vitro* human model to study pathophysiological processes involved in human AA is lacking. For this reason, we developed a method to obtain vital, *ex vivo* human vascular tissue. In these cultured aneurysmal and non-aneurysmal vascular specimens, evaluation of viability showed that the larger part of human vascular tissue sections can be kept alive in culture for up to 62 days, while maintaining microstructural organisation of the vascular wall. The live cells were mainly observed in the centre of human tissue. As expected, dead cells were mainly located at the edges of the tissue, which were subjected to cutting trauma. After optimization of the protocol, we attained even higher proportions of surviving tissue.

Interestingly, viability proportion outcomes increased after day 3 compared to day 0. These results are strengthened by not only the significant difference in mean viability of tissue sections between day 0 and day 14, but also between day 5 and day 14. Cells might go through an early phase of apoptosis due to stress factors arising from physical trauma. Initially, these mechanisms might still be reversible^17,18^, with subsequent recovery as of day 3. Another possible explanation for the observed increase of live cells is cell proliferation. This theory is supported by the development and expansion of live cells, observed in the tissues cultured over 60 and 90 days and the growth of new cells at the intimal side.

Another important finding was that we achieved to preserve tissue architecture and infiltrating cells *ex vivo*. In this composition, we found many live smooth muscle cells as the main cell type, but we also observed leucocytes and macrophages involved in the occurrence and aggravation of AA^19–21^.^.^ Based on our composition staining using smoothelin and phalloidin, we provide support for our hypothesis that both tunica media and tunica adventitia are preserved after 14 days. Phalloidin is not specific for fibroblasts. However, phalloidin staining in in spindle shaped cells combined with lack of smoothelin staining in the lower layer of the tissue sections, strongly indicates fibroblasts, which are generally known to be in the tunica adventitia. Quantification results showed an increase in fluorescence per nucleus in both smoothelin and phalloidin. This might implicate, that although over time the viability diminishes, the actual cell size, or expression of cell markers increases over time. However, based on the results of one study patient, caution must be applied, as the findings might not be representative for other patients. Unfortunately, the friable tunica intima is frequently damaged due to vibratome sectioning. Preservation of the AA tissue composition in *ex vivo* tissues facilitates the study of cell behaviour and interaction between different cell types, and provides the opportunity to investigate the effects of pharmacological substances on tissue composition or in organ on a chip models.

Analysis of digested tissue sections showed a high viability of single cells, even after treatment with collagenase. Again, different cell types could be morphologically distinguished, supporting the purpose of this protocol; creating an *ex vivo* arterial environment, which mimics the *in vivo* interaction of different cell types and extracellular matrix. The ability to digest the tissue sections greatly broadens the possibilities of this protocol. For example, FACS, MACS and traction force microscopy are examples of downstream applications that can be performed to expand the characterisation of cells which have been cultured and potentially stimulated in an *ex vivo* aortic microenvironment^22–24^.

To evaluate the feasibility of live aortic tissue section stimulation and RNA expression analysis, we stimulated aortic vibratome tissue sections with TGF-β, since prior studies have provided paradoxical hypotheses on the role of TGF-β (and thereby stimulation by angiotensin II) in the development and progression of AAA^25,26^. In this pooled stimulated versus non-stimulated analysis, we found elevated RNA expression of interleukin 6 *(IL6)*, calponin 1 *(CNN1)* and transforming growth factor beta 1 *(TGFB1)*, in the stimulated tissue sections. Interestingly, TGF-β has previously been reported to elevate expression of interleukin 6 in different types of fibroblasts, which is in line with our results^27,28^. Simultaneously, it has been previously observed, that TGF-β overexpression in animal models decreases MMP2, MMP9 and lymphocytes (PTPRC), consistent with our findings in human tissue sections^29^. However, with a small sample size and pooled patient data, caution must be applied, as the findings might not be representative for all AAA patients. Future studies with the current method are therefore recommended.

The results of this study meet the need for a new *in vitro* model in which the pathophysiological processes involved in human AA can be studied. In our protocol, sectioning is performed using the vibratome, which has been successfully used for the live cutting and *ex vivo* preservation of cardiac mouse tissues and human salivary gland and tumour sections of breast and parathyroid for a maximum of two weeks^14,30–33^. However, this is the first report of a protocol demonstrating over 60 days *ex vivo* viability in vibratome sectioned aortic tissue.

It can be argued that cells with a short lifespan or cells vulnerable to culturing will not survive for a long period in the *ex vivo* tissue sections. Nevertheless, during the first days of culturing we can study the individual cells, interactions between cells and ECM, behavior of immune cells and remodelling of the tissue as seen in other specialist fields using vibratome sections^25^. Subsequently, we have successfully stimulated the live tissue sections and the live tissue can serve as a patient specific scaffold on which new cells can be seeded and pharmacological experiments can be performed.

To the best of our knowledge, we are the first to present a method to keep entire aortic tissue sections alive *in vitro*. This method may be applicable also to other human (cardiovascular) tissue. We achieved slicing, preservation, proliferation, and analysis of live aortic tissues for at least 60 days after harvesting. Diverse cell types (including smooth muscle cells and white blood cells) and intracellular characteristics of live tissues were presented. Besides well-known fluorescence imaging, FACS, MACS and traction force microscopy, we are currently investigating the use of these tissue sections in new techniques in which we study elastic properties, aortic wall secretome, and pathways by additional inhibition/stimulation tests. By having established a method for extended *in vitro* preservation of functionally and structurally intact vascular tissue sections, research on the etiopathophysiology of AA and possibly other vascular diseases may have entered a new era.

## Electronic supplementary material

Supplementary Table S1. RefSeq codes and primer sequences
