# Supplementary material for: An in vitro method to keep human aortic tissue sections functionally and structurally intact
Source: Sci Rep. 2018 May 25;8:8094. doi: 10.1038/s41598-018-26549-4 (PMC5970251; doi:10.1038/s41598-018-26549-4)
Supplement: Supplementary file 1 — Supplementary Table S1. RefSeq codes and primer sequences [file 41598_2018_26549_MOESM1_ESM.docx]

**An in vitro method to keep human aortic tissue sections functionally and structurally intact**

Jorn P. Meekel, MD^1,2^; Menno E. Groeneveld, MD^1,2^; Natalija Bogunovic; MSc^1,2^; Niels Keekstra, BSc^1^; René J.P. Musters, MSc, PhD^2^; Behrouz Zandieh-Doulabi, MSc, PhD^3^; Gerard Pals, MSc, PhD^3^; Dimitra Micha, MRes, PhD^3^; Hans W.M. Niessen, MD, PhD^4^; Arno M. Wiersema, MD, PhD^5^; Jur K. Kievit, MD^5^; Arjan W.J. Hoksbergen, MD, PhD^1^; Willem Wisselink, MD, PhD^1^; Jan D. Blankensteijn, MD, PhD^1^ and Kak K. Yeung, MD, PhD^1,2*^.

| Gene name | RefSeq | Forward and reverse primer sequence |
| --- | --- | --- |
| *YWHAZ* | [NM_145690](http://www.ncbi.nlm.nih.gov/nuccore/NM_145690) | GATGAAGCCATTGCTGAACTTG |
|  |  | CTATTTGTGGGACAGCATGGA |
| *IL6* | [NM_000600](http://www.ncbi.nlm.nih.gov/nuccore/NM_000600) | ACAGCCACTCACCTCTTCA |
|  |  | ACCAGGCAAGTCTCCTCAT |
| *CNN1* | [NM_001308341](http://www.ncbi.nlm.nih.gov/nuccore/NM_001308341) | GCCCAGAAGTATGACCACCA |
|  |  | TGATGAAGTTGCCGATGTTC |
| *TGFB1* | [NM_000660](http://www.ncbi.nlm.nih.gov/nuccore/NM_000660) | CCGACTACTACGCCAAGGAG |
|  |  | GGTATCGCCAGGAATTGTTG |
| *MCP1* | [NM_002982](http://www.ncbi.nlm.nih.gov/nuccore/NM_002982) | CACTCTCGCCTCCAGCATGA |
|  |  | ATCTCCTTGGCCACAATGGT |
| *TGFBR1* | [NM_001306210](http://www.ncbi.nlm.nih.gov/nuccore/NM_001306210) | ACAACCGCACTGTCATTCAC |
|  |  | TCTCCAAACTTCTCCAAATCG |
| *SMTN* | [NM_001207018](http://www.ncbi.nlm.nih.gov/nuccore/NM_001207018) | TGGAGGAATTGACTGCACTG |
|  |  | GAAACCTCTGCCTGCTGTTC |
| *ICAM3* | [NM_001320605](http://www.ncbi.nlm.nih.gov/nuccore/NM_001320605) | TGTTTGAAGGAAGGCTCCAG |
|  |  | AGTAACACCGCCACGAAGAC |
| *MMP2* | [NM_004530](http://www.ncbi.nlm.nih.gov/nuccore/NM_004530) | CCGTGGTGAGATCTTCTTCTTC |
|  |  | GCTGGTCAGTGGCTTGGGGTA |
| *ACTA2* | [NM_001141945](http://www.ncbi.nlm.nih.gov/nuccore/NM_001141945) | ACTGGGACGACATGGAAAAG |
|  |  | CATACATGGCTGGGACATTG |
| *TNF* | [NM_000594](http://www.ncbi.nlm.nih.gov/nuccore/NM_000594) | TCAACCTCCTCTCTGCCATC |
|  |  | AGTAGACCTGCCCAGACTCG |
| *PTPRC* | [NM_080921](http://www.ncbi.nlm.nih.gov/nuccore/NM_080921) | AGTATCCCCGGACTCTTTGG |
|  |  | AGGGTTGAGTTTTGCATTGG |
| *Ki67* | [NM_002417](http://www.ncbi.nlm.nih.gov/nuccore/NM_002417) | AGCACCAGAGGAAATTGTGG |
|  |  | TTTTCAGGGACCGAGTCTTG |
| *ICAM1* | [NM_000201](http://www.ncbi.nlm.nih.gov/nuccore/NM_000201) | GGCTGGAGCTGTTTGAGAAC |
|  |  | AGGAGTCGTTGCCATAGGTG |
| *IL8 (CXCL8)* | [NM_000584](http://www.ncbi.nlm.nih.gov/nuccore/NM_000584) | GTGTGAAGGTGCAGTTTTGC |
|  |  | CCTTGGGGTCCAGACAGAG |
| *MMP9* | [NM_004994](http://www.ncbi.nlm.nih.gov/nuccore/NM_004994) | ACAGGCAGCTGGCAGAGGA |
|  |  | CGCGGCAAGTCTTCCGAGTA |

**Supplementary Table S1. RefSeq codes and primer sequences**

RefSeq code and forward and reverse primer sequences per corresponding gene. RefSeq indicates NCBI Reference Sequence Database code.
